# Skin model for improving the reliability of the modified Rodnan skin score for systemic sclerosis

**DOI:** 10.1186/s41927-022-00262-2

**Published:** 2022-06-02

**Authors:** Patnarin Pongkulkiat, Bandit Thinkhamrop, Ajanee Mahakkanukrauh, Siraphop Suwannaroj, Chingching Foocharoen

**Affiliations:** 1 Department of Medicine, Faculty of Medicine, Khon Kaen University, Khon Kaen, 40002 Thailand; 2 Department of Epidemiology and Biostatistics, Faculty of Public Health, Khon Kaen University, Khon Kaen, 40002 Thailand

**Keywords:** Scleroderma and related disorders, Skin, Clinical trials and methods, Primary care rheumatology, Study design

## Abstract

**Background:**

The gold standard for skin thickness assessment in systemic sclerosis (SSc) is the modified Rodnan skin score (mRSS); however, inter- and intra-rater variation can arise due to subjective methods and inexperience. The study aimed to determine the inter- and intra-rater variability of mRSS assessment using a skin model.

**Methods:**

A comparative study was conducted between January and December 2020 at Srinagarind Hospital, Khon Kaen University, Thailand. Thirty-six skin sites of 8 SSc patients underwent mRSS assessment: 4 times the first day and 1 time over the next 4 weeks by the same 10 raters. No skin model for mRSS assessment was used for the first two assessments, while one was used for the remaining three rounds of assessments. The Latin square design and Kappa statistic were used to determine inter- and intra-rater variability.

**Results:**

The kappa agreement for inter-rater variability improved when the skin model was used (from 0.4 to 0.5; 25%). The improvement in inter-rater variability was seen in the non-expert group, for which the kappa agreement rose from 0.3 to 0.5 (a change of 66.7%). Intra-rater variability did not change (kappa remained at 0.9), and the long-term effect of using a skin model slightly decreased by week 4 (Δkappa 0.9–0.7).

**Conclusions:**

Using a skin model could be used to improve inter-rater variation in mRSS assessment, especially in the non-expert group. The model should be considered a reference for mRSS assessment in clinical practice and health education.

**Supplementary Information:**

The online version contains supplementary material available at 10.1186/s41927-022-00262-2.

## Background

Systemic sclerosis (SSc) is a complex multisystem autoimmune connective tissue disease. SSc has been classified into two subtypes: (1) limited cutaneous systemic sclerosis (lcSSc) in which skin thickness is limited, presenting distal to the elbows and knees, with or without face involvement, and (2) diffuse cutaneous systemic sclerosis (dcSSc) in which the extent of skin involvement presents above the elbows and knees, with or without face involvement [1]. The most common symptom and cause for concern among SSc patients is skin thickening [2].

The assessment of severity and extent of skin thickness is crucial as it is a surrogate marker of disease activity, severity, and prognosis as well as treatment responsiveness. The methods for skin thickness assessment thus need to be valid, reliable, precise, and practicable [3–6].

The skin biopsy validated, gold standard for skin thickness assessment for SSc is the modified Rodnan skin score (mRSS) [7–12]. The mRSS assesses skin thickness from 17 body sites: the face, chest, abdomen, arms, forearms, hands, fingers, thighs, legs, and feet. A score of 0 indicates normal skin thickness, 1 mild skin thickness, 2 moderate skin thickness, and 3 severe skin thickness with an inability to make skin folds between two fingers. The score is calculated by summing the rating from all 17 areas (range 0–51) [13, 14].

Although the mRSS has been validated at many centers, it has some limitations such as significant inter- and intra-rater variability due to (a) its subjective methodology, (b) physician inexperience, (c) significant differences between ethnic groups, (d) inaccuracies during the edematous and atrophic skin phase, and (e) lack of sensitivity in measuring minimal changes [7, 15–17].

To overcome these limitations, researchers have tried to develop new objective and quantitative methods for skin assessment. Mechanical devices and new imaging techniques include the durometer—a handheld device that measures skin hardness [18–20]; the plicometer—a medical device that measures skin folding [21]; the cutometer—a device that measures skin elasticity [22, 23]; the vesmeter—a computer-linked device that measures skin hardness, elasticity, and viscosity [24]; the twistometer—a device that measures skin rotation [25]; high-frequency ultrasound—an objective and quantitative tool that measures skin thickness [26, 27]; elastosonography—a tool that measures skin elasticity [28, 29]; shear wave elastography—a tool that measures skin thickness [30]; magnetic resonance imaging—a tool for demonstrating abnormalities of the skin and subcutaneous tissues [31, 32]; and, optical coherence tomography—a tool that identifies the microscopic features of the skin [33–35]. None of these techniques has matched the OMERACT standard of the mRSS for assessing the validity of outcomes; usually, because the techniques are not feasible in clinical practice due to time constraints, accessibility, dependence on trained experts, or lack of clarity defining what aspect of the skin to assess [8, 36].

Since mRSS is a validated outcome in scleroderma, specialist rater training was needed to improve accuracy and reduce variability. Limitations of the mRSS skin assessment include; (a) training of the mRSS skin assessment needs experienced rheumatologist as a trainer, the training process might be affected if there are limited numbers of experienced rheumatologist; (b) the mRSS assessment method takes time and the trainee needs a learning curve, and; (c) subjective skin assessment according to the mRSS method needs recall memory, so it might affect the accuracy of data and causes a recall bias. The idea of using a skin model arose as a way to address these limitations. Trainees can use this skin model, which has been validated by experienced rheumatologists, as a reference for skin thickness severity assessment without needing to take specialist skin assessment training from a rheumatologist and using recall memory after training. Moreover, nurses and/or healthcare workers can also perform skin assessments using the skin model as a reference. If the skin model can be validated, it would be help ensure initial early disease severity assessments, early management, and early referral to specialists. In addition, using the skin model might save resources and provide better care for SSc patients.

The skin model has four grades of skin thickness, just like the mRSS (viz., 0, 1, 2, and 3 as validated by experienced rheumatologists) [14, 37]. The model is used as a nonviable trainer for inexperienced physicians who palpate each site of the patient’s skin, compare it to the model, and score according to the mRSS assessment method. The study's objectives were to determine the inter- and intra-rater variability of the mRSS assessment after using the skin model. If the model achieved good agreement vis-à-vis both inter- and intra-rater variabilities for inexperienced or non-expert assessors, the model could be used as a reference of skin thickness assessment as per the mRSS assessment method for health education, routine clinical practice, and/or clinical trials.

## Methods

A descriptive, comparative, reliability study was conducted on eight Thai adult SSc patients, followed up at the Scleroderma Clinic, Srinagarind Hospital, Khon Kaen University, between January and December 2020.

**Fig. 1:**
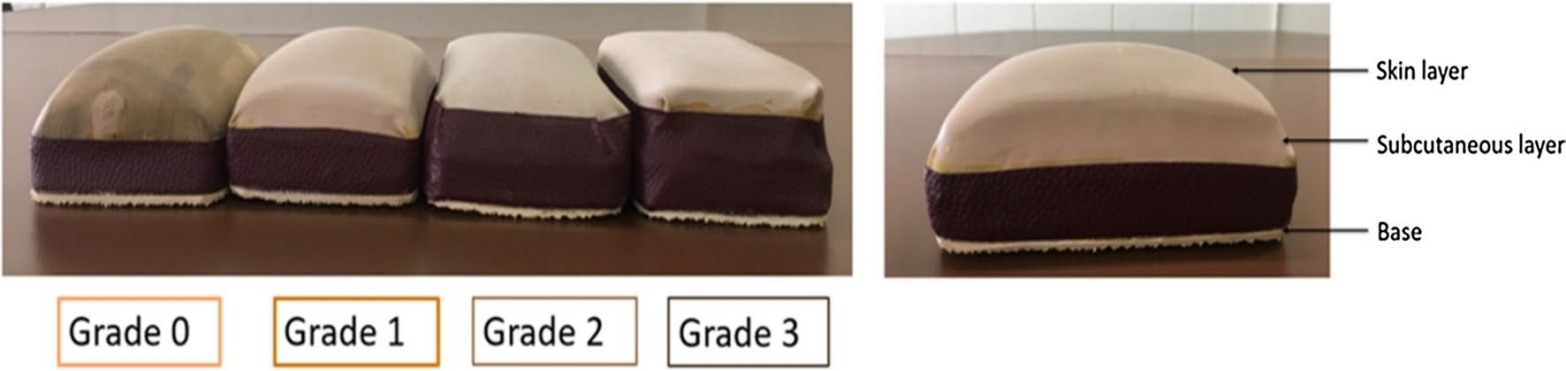
Skin model

The skin model was developed to mimic the four grades of disease according to the mRSS. The model comprises three layers—the base is made from polypropylene to maintain structural integrity; the subcutaneous layer from a synthetic sponge with or without polyurethane foam; and, the skin layer from raw, skin-colored, or synthetic rubber. The density of the polyurethane foam is set by the degree of skin elasticity being modeled: Grade 0—sponge alone; Grade 1—low-density polyurethane foam superimposed on a synthetic sponge; Grade 2—medium density polyurethane foam superimposed on a synthetic sponge; and Grade 3—high-density polyurethane foam superimposed on the synthetic sponge. According to the skin grading, the thickness of the skin layer is adjusted to be between 1 and 5 mm (Fig. 1). Four experienced rheumatologists independently validated the model. Each grade of the skin model was labeled after validation.

A Latin square experiment was used to determine the inter- and intra-rater variability of mRSS using the skin model. The sample size was calculated based on the kappa agreement to quantify the reliability among ten raters doing an mRSS assessment (range, 0–3). For example, a sample size of 36 skin sites (9 sites for each mRSS grading severity) with ten raters per subject would achieve 80% power to detect a kappa agreement of 0.80 under the alternative hypothesis when the kappa agreement under the null hypothesis is 0.49 (probability 0.25, 0.30, 0.30, 0.15) using an F-test with a significance level of 0.05.

All patients were over 18 years of age and met the 1980 American Rheumatism Association classification criteria of SSc [38] or 2013 ACR/EULAR Classification Criteria for Scleroderma [39]. Patients were excluded if they had any of the following conditions: overlap syndrome, a WHO functional class IV, needing oxygen therapy at rest, not available for skin assessment (i.e., post-amputation status), recent soft tissue or skin infection, coexisting disease including cancer, severe sepsis, and/or psychiatric or neurological problems.

Ten raters were included in the study, including two rheumatologists, three rheumatology fellows, three internal medicine residents, and two nurses. The types of raters were divided into expert raters (2 rheumatologists and 3 rheumatology fellows) and non-expert raters (3 general practitioners and 2 nurses). The 36 skin sites (9 sites for each level of mRSS severity grading ranging from 0 to 3) were selected and marked on 8 patients by consensus of 2 experienced rheumatologists.

**Fig. 2:**
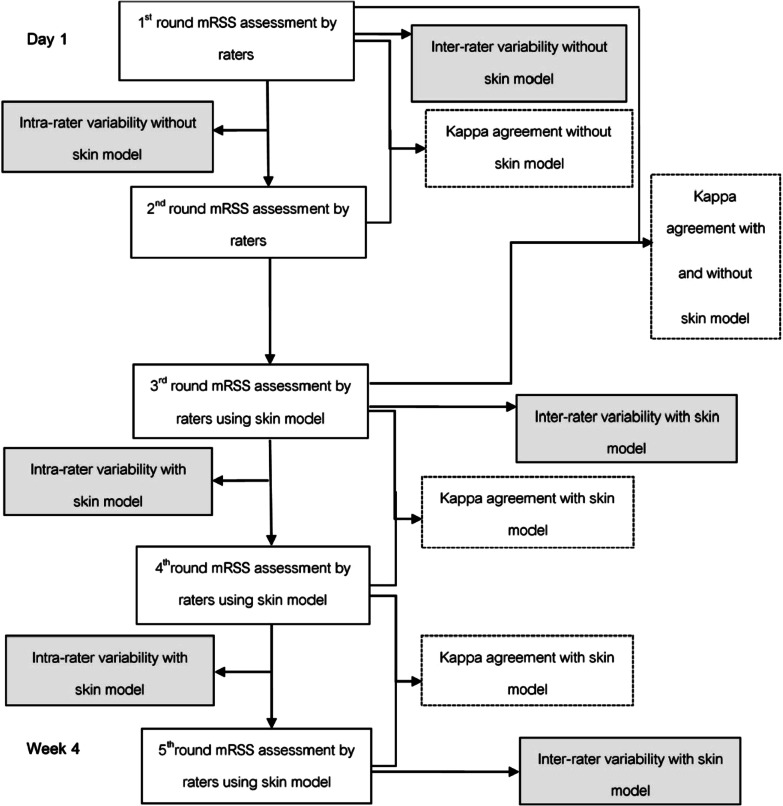
Study flow chart

An experienced rheumatologist trained all raters on mRSS assessment before performing any assessments. Five assessments were performed. Each rater assessed 36 predetermined skin sites per session. The first and second assessments were performed about 30 min apart without using a skin model. The third and fourth assessments were performed 30 min apart using the skin model as the reference. The first four assessments were performed on day 1 of the study. The fifth assessment using the model was done 4 weeks later (Fig. 2). The assessments were independent and blinded from each rater.

### Statistical analysis

The demographic characteristics are presented using means (± SD) for the continuous data and numbers with percentages for the categorical data.

In the Latin square experiment, inter- and intra-rater variability of mRSS with and without using the skin model were assessed using the kappa statistic, a statistic used to measure inter- and intra-rater agreement for categorical items by eliminating agreement by chance. The kappa with its 95% confidence interval was estimated to demonstrate the level of agreement.

The first two assessments were used to investigate intra-rater reliability absent any interventions. The first assessment score was compared with the third assessment to investigate the intra-rater reliability under the rating with and without the skin model. The third and fourth assessments investigated intra-rater reliability under the rating using the skin model as the reference. The final kappa agreement comparing the score between the last two assessments 4 weeks apart was to evaluate the skin model's sustained improvement vis-à-vis inter-rater reliability. The kappa agreement was also evaluated to determine inter-rater reliability according to the experts in the mRSS assessment (expert vs. non-expert raters). The overall difference in kappa agreement between the first and fourth assessments with and without the skin model reflects the effect of using the skin model.

All data analyses were performed using STATA version 16.0 (StataCorp., College Station, TX, USA) and R program version 4.0.3.

The Human Research Ethics Committee of Khon Kaen University approved the study as per the Helsinki Declaration and the Good Clinical Practice Guidelines (HE621504). The experiment was explained to all participants who then signed informed consent before enrollment.

## Results

### Patients and raters baseline characteristics

The study included 8 SSc patients (5 males). The mean age was 59.0 ± 3.5 years. Both SSc subsets were included (7 dcSSc and 1 lcSSc). The mean disease duration was 5.8 ± 3.9 years. The ten raters included 7 females whose mean age was 33.7 ± 7.7 years (Table 1).

**Table 1:** Demographics of patients and raters presented as number and percentage unless specified otherwise

Characteristic	Number N (%)
*A. Patient characteristics*	N = 8
Age in years, mean** ± **SD (min–max)	59.0 ± 3.5 (55–64)
Duration of disease in years, mean** ± **SD (min–max)	5.8 ± 3.9 (0.7–13.9)
Sex female	3 (37.5)
Male	5 (62.5)
Subset dcSSc	7 (87.5)
lcSSc	1 (12.5)
*B. Rater characteristics*	N = 10
Age in years, mean ± SD (min–max)	33.7 ± 7.7 (25–48)
Sex: female	7 (70)
Male	3 (30)
Type of raters	
Rheumatologists	2 (20)
Fellowship in rheumatology	3 (30)
Internists	3 (30)
Nurse	2 (20)

*SD* standard deviation

### Skin score assessment

The respective individual kappa agreement for the intra-rater variability analysis with and without the skin model was 0.9 and 0.9 (Table 2.); however, the intra-rater agreement variability using the skin model at week 4 decreased slightly to 0.7 (Table 2).

**Table 2:** Kappa agreement for intra-rater variability of skin thickness scoring agreement

	Agreement (%)	Overall kappa	95%CI
Assessment without skin model (round 1 and 2)	96.1	0.9	0.82–0.98
Assessment with skin model (round 3 and 4)	95.4	0.9	0.82–0.98
Assessment with skin model (round 4 and 5)	89.8	0.7	0.62–0.78

*CI* confidence interval

The respective overall kappa agreement for inter-rater variability analysis with and without the skin model was 0.5 and 0.4, respectively (25% difference). Notwithstanding, the kappa agreement for inter-rater variability analysis in the non-expert group was improved by 67% when using the skin model compared to not using the skin model (kappa = 0.3 vs. 0.5), while the kappa agreement was comparable in the expert group with and without the skin model (kappa = 0.5 vs. 0.5). (Table 3.)

**Table 3:** kappa agreement for inter-rater variability analysis in overall raters, expert-raters, and non-expert raters

	Overall kappa	95%CI	Kappa of expert	95%CI	Kappa of non-expert	95%CI
Assessment without skin model	0.4	0.39–0.41	0.5	0.47–0.53	0.3	0.27–0.33
Assessment skin model	0.5	0.49–0.51	0.5	0.47–0.53	0.5	0.47–0.53
Percentage of difference	25.0%	–	0%	–	66.7%	–

*CI* confidence interval

The overall and individual skin thickness agreement in day 1 and week 4 are presented in Additional file 1: Table S1, Additional file 2: Table S2, Additional file 3: Table S3, Additional file 4: Table S4, Additional file 5: Table S5, Additional file 6: Table S6 and Additional file 7: Table S7.

## Discussion

Skin thickness is the most troubling clinical symptom among SSc patients. In SSc, the extent and severity of skin thickness are also associated with the severity of internal organ involvements, poor prognosis, morbidity, and mortality [2–6]. The accuracy and validity of mRSS, the gold standard for skin thickness assessment, are essential [7, 8, 10]. Although previous studies revealed that the mRSS was a valid and reliable test for skin thickness assessment, there is significant inter- and intra-rater variation, particularly when inexperienced assessors perform it [10, 15, 16]. A similar finding was shown in a study by Foocharoen et al. conducted among Thai SSc patients [17]. To overcome this limitation, we developed a skin model to use as a reference for mRSS assessment to reduce inter- and intra-rater variability in mRSS assessment.

We found that the overall inter-rater variability (kappa agreement) of the mRSS assessment when using the skin model improved from 0.4 to 0.5—a 25% improvement. In the non-expert group, where the kappa agreement had been 66.7%, agreement rose from 0.3 to 0.5. By comparison, in the expert group, the inter-rater agreement did not improve (before 0.5 and after 0.5). Our results agree with previous studies where mRSS assessment accuracy improved with experience [15, 16, 40, 41]. The inter-rater variability for mRSS assessment using the skin model was good compared to raters who took a standardized mRSS training course [40]. The EUSTAR study (European League Against Rheumatism Scleroderma Trials and Research) reported that repeated mRSS assessment training courses decrease inter-rater variability. The ICC (intra-class correlation coefficient) agreement increased from 0.5 to 0.7 after two training courses in less experienced rheumatologists, while for experts it was very good at the outset and did not increase [42]. The authors also found that intra-rater variability was relatively good before mRSS assessment training and remained stable thereafter [42]. Another report reported a significant difference in the training effect according to physicians’ professional seniority. The training effect was more pronounced in students than in senior staff [40]. The results of our study likewise showed good intra-rater agreement that did not change when using the skin model. In addition, using a skin model can save time, no need to recall memory, no need learning curve, and the mRSS assessment training by the specialist. Our results suggest that the skin model might be used as a reference for mRSS assessment instead of a health education training course.

Intra-rater variability doing the mRSS assessment with or without the skin model was comparable on day 1 (pre-/post kappa were both 0.9), perhaps because of a post-training effect lending confidence on how to assess skin thickness using mRSS. After evaluating the long-term effects on intra-rater variability, the kappa agreement was slightly dropped from 0.9 on the first day of evaluation to 0.7 by week 4, even though skin thickness had hardly progressed over the 4-week study period. The results suggest that the time interval might affect intra-rater variability. Although there was a slight increase in long-term intra-rater variability, the inter-rater variability was not much improved. The findings suggest a decline in rater confidence in only 1 round of skin assessment, week 4, without training or orientation. Further study is suggested to evaluate intra-rater variability over a longer duration to confirm the long-term effect on intra-rater variability when using the skin model. We propose doing further study, including internal and external validation of the skin model vis-à-vis implementation as a reference for mRSS assessment in daily practice and/or clinical trials.

Our study had some limitations, including (1) we are a single-center trial, so further investigation is needed of the external validity of the test, and (2) we enrolled only Asians whose skin thickness may be different from other ethnic groups [10, 43]. Notwithstanding, the reliability of mRSS assessment does not depend on the patients' skin rather it depends upon the assessors. Irrespective of the patient's nationality or ethnicity, the skin assessment is unlikely to influence the findings. Although a small number of patients (8 patients with 36 skin sites) were included in determining inter- and intra-rater variability of mRSS using the skin model, we are confident that we have included an adequate sample size according to the method of sample size calculation. Further study for external validation of the skin model should include a larger sample size.

The strengths of our study are that (1) we included both dcSSc and lcSSc, both sexes, various ages, various disease durations, and various sites of skin assessment in SSc which can represent general SSc patient and can be generalized; (2) both expert and non-expert raters were included, so the results can be implemented in daily practice and/or in clinical trial(s) if experienced rheumatologists or specialists are unavailable; (3) the skin assessment was performed 4 times on day 1 and one more time during week 4 (i.e., 2 times without the skin model and 3 times with the skin model). We thus have confidence that we have investigated both inter- and intra-rater variability; and (4) we used materials that are versatile, low-cost, available, and nontoxic. The textiles are like human skin, so the skin model can be easily produced, is harmless and long-lasting.

## Conclusion

The skin model improves inter-rater reliability of mRSS assessment, especially in the non-expert group. This finding suggests that the skin model might be helpful as a reference for mRSS assessment training and in clinical practice. However, further study is needed to assess the validity and the sustained effect in a larger population.

## Supplementary Information

**Additional file 1.**  Overall skin thickness scoring agreement without skin model on day 1.

**Additional file 2.**  Overall skin thickness scoring agreement with and without skin model on day 1.

**Additional file 3.** Overall skin thickness scoring agreement with skin model on day 1.

**Additional file 4.** Overall skin thickness scoring agreement with skin model between round 4 and 5.

**Additional file 5.** Individual skin thickness scoring agreement without skin model (1st and 2nd round).

**Additional file 6.**  Individual skin thickness scoring agreement with skin model (3rd and 4th round).

**Additional file 7.** Individual skin thickness scoring agreement with skin model (4th and 5th round).

## Data Availability

The datasets used and/or analysed during the current study are available from the corresponding author on reasonable request.

## Abbreviations

SSc: Systemic sclerosis
lcSSc: Limited cutaneous systemic sclerosis
dcSSc: Diffuse cutaneous systemic sclerosis
mRSS: Modified Rodnan skin score
