# Supplementary material for: Skin model for improving the reliability of the modified Rodnan skin score for systemic sclerosis
Source: BMC Rheumatol. 2022 Jun 2;6:33. doi: 10.1186/s41927-022-00262-2 (PMC9161481; doi:10.1186/s41927-022-00262-2)
Supplement: Supplementary file 2 — Additional file 2. Overall skin thickness scoring agreement with and without skin model on day 1. [file 41927_2022_262_MOESM2_ESM.docx]

**Additional file 2**

**Table S2.** Overall skin thickness scoring agreement with and without skin model on day 1

| 3^rd^ round skin thickness assessment with skin model | 1^st^ round skin thickness assessment without skin model | | | | |
| --- | --- | --- | --- | --- | --- |
|  | Score | 0 | 1 | 2 | 3 |
|  | 0 | 62 | 2 | 0 | 0 |
|  | 1 | 30 | 28 | 7 | 0 |
|  | 2 | 1 | 39 | 50 | 12 |
|  | 3 | 0 | 2 | 27 | 100 |
