# Supplementary material for: Skin model for improving the reliability of the modified Rodnan skin score for systemic sclerosis
Source: BMC Rheumatol. 2022 Jun 2;6:33. doi: 10.1186/s41927-022-00262-2 (PMC9161481; doi:10.1186/s41927-022-00262-2)
Supplement: Supplementary file 4 — Additional file 4. Overall skin thickness scoring agreement with skin model between round 4 and 5. [file 41927_2022_262_MOESM4_ESM.docx]

**Additional file 4**

**Table S4.** Overall skin thickness scoring agreement with skin model between round 4 and 5

| 5^th^ round skin thickness assessment with skin model at week 4 | 4^th^ round skin thickness assessment with skin model at day 1 | | | | |
| --- | --- | --- | --- | --- | --- |
|  | Score | 0 | 1 | 2 | 3 |
|  | 0 | 47 | 11 | 4 | 0 |
|  | 1 | 15 | 40 | 27 | 0 |
|  | 2 | 0 | 10 | 53 | 28 |
|  | 3 | 0 | 1 | 9 | 115 |
