# Supplementary material for: Skin model for improving the reliability of the modified Rodnan skin score for systemic sclerosis
Source: BMC Rheumatol. 2022 Jun 2;6:33. doi: 10.1186/s41927-022-00262-2 (PMC9161481; doi:10.1186/s41927-022-00262-2)
Supplement: Supplementary file 6 — Additional file 6. Individual skin thickness scoring agreement with skin model (3rd and 4th round). [file 41927_2022_262_MOESM6_ESM.docx]

**Additional file 6**

**Table S6.** Individual skin thickness scoring agreement with skin model (3^rd^ and 4^th^ round)

| **Rater** | **Agreement** | **Kappa** |
| --- | --- | --- |
| **1** | 100.0% | 1.0 |
| **2** | 100.0% | 1.0 |
| **3** | 83.3% | 0.8 |
| **4** | 75.0% | 0.6 |
| **5** | 77.8% | 0.7 |
| **6** | 80.6% | 0.7 |
| **7** | 80.6% | 0.7 |
| **8** | 100.0% | 1.0 |
| **9** | 83.3% | 0.8 |
| **10** | 80.6% | 0.7 |
| **Overall** | 95.4% | 0.9 |
