# Supplementary material for: Skin model for improving the reliability of the modified Rodnan skin score for systemic sclerosis
Source: BMC Rheumatol. 2022 Jun 2;6:33. doi: 10.1186/s41927-022-00262-2 (PMC9161481; doi:10.1186/s41927-022-00262-2)
Supplement: Supplementary file 7 — Additional file 7. Individual skin thickness scoring agreement with skin model (4th and 5th round). [file 41927_2022_262_MOESM7_ESM.docx]

**Additional file 7**

**Table S7.** Individual skin thickness scoring agreement with skin model (4^th^ and 5^th^ round)

| **Rater** | **Agreement** | **Kappa** |
| --- | --- | --- |
| **1** | 86.1% | 0.8 |
| **2** | 94.4% | 0.9 |
| **3** | 61.1% | 0.5 |
| **4** | 75.0% | 0.6 |
| **5** | 61.1% | 0.4 |
| **6** | 63.9% | 0.5 |
| **7** | 77.8% | 0.7 |
| **8** | 69.4% | 0.5 |
| **9** | 55.6% | 0.4 |
| **10** | 63.9% | 0.5 |
| **Overall** | 89.8% | 0.7 |
